# High-speed videography of transparent media using illumination-based multiplexed schlieren

**DOI:** 10.1038/s41598-022-23198-6

**Published:** 2022-11-08

**Authors:** Simon Ek, Vassily Kornienko, Adrian Roth, Edouard Berrocal, Elias Kristensson

**Affiliations:** Division of Combustion Physics, Lund University, Professorsgatan 1, Lund, Sweden

**Keywords:** Imaging and sensing, Imaging techniques

## Abstract

Schlieren photography is widely used for visualizing phenomena within transparent media. The technique, which comes in a variety of configurations, is based on detecting or extracting the degree to which light is deflected whilst propagating through a sample. To date, high-speed schlieren videography can only be achieved using high-speed cameras, thus limiting the frame rate of such configurations to the capabilities of the camera. Here we demonstrate, for the first time, optically multiplexed schlieren videography, a concept that allows such hardware limitations to be bypassed, opening up for, in principle, an unlimited frame rate. By illuminating the sample with a rapid burst of uniquely spatially modulated light pulses, a temporally resolved sequence can be captured in a single photograph. The refractive index variations are thereafter measured by quantifying the local phase shift of the superimposed intensity modulations. The presented results demonstrate the ability to acquire a series of images of flame structures at frame rates up to 1 Mfps using a standard 50 fps sCMOS camera.

## Introduction

Schlieren is an imaging technique, invented more than one century ago, that allows faint refractive index variations in transparent media to be visualized^1–4^. With time, alternative optical configurations with similar imaging capabilities have emerged, all of which rely on detecting the refraction that occurs at the interface between regions of different refractive index. Such variations can be caused by changes in pressure, temperature or constituents, allowing phenomena such as shock-waves^5–7^, heat transfer^8,9^, combustion^10,11^ and gas flow^12,13^ to be studied.

To visualize the variations in refractive index, the fundamental schlieren configuration is based on transmission imaging with a collimated light source followed by spatial filtering to optically reject part of the light, according to degree of deflection^2,14^. Such a configuration thus permits direct visualization of regions within the sample where the light was refracted. In 1949, Burton et al.^15^ demonstrated the Moiré Fringe Method (MFM) as an alternative schlieren configuration wherein two optically miss-matched transmission gratings are placed on either side of the object, thus blocking non-deflected light whilst permitting photons that deviate from the original trajectory. The benefit with MFM over conventional schlieren is that it can provide a larger field-of-view (FOV). More recently, Background Oriented schlieren (BOS)^4,16–18^ has emerged as an attractive alternative to schlieren imaging, having a reduced experimental complexity compared to illumination-based schlieren. Instead of optically rejecting light that does not deviate from the original trajectory, BOS uses a textured background. Light refraction yields local distortions in this texture and by quantifying the distortion the refractive index gradients are deduced. Any textured—random^17^, periodic^19^ or structured^20^—background can be used, allowing e.g. supersonic air-craft shock waves to be observed by using the textured ground as background^16^. In an early version of BOS, labeled synthetic schlieren, a grating was used as background, but unlike the closely related MFM this technique utilizes an undistorted image of the background as a reference, instead of a second grating^21^. The technique shares a common limitation with schlieren and MFM in that it can only detect refractive index gradients perpendicular to the mask/grating edges. Fast Checkerboard Demodulation (FCD), demonstrated by Wildeman in 2018, is a way of overcoming this limitation, by using a checkered background and subsequent Fourier demodulation of the captured image^20^. The technique produces two unique images, corresponding to refractive index gradients in x- and y-direction, respectively, thus allowing the full 2D vector displacement field to be calculated.

Schlieren imaging does not require any particular detector. Thus, by utilizing high-speed sensor technology and intense illumination sources, rapid transient events can be studied with schlieren^4^. High-speed schlieren videography in the $10^3$–$10^6$ Hz video rate range can be achieved using fast sensor technology^22–25^ while, with current technology, faster video rates require multiple intensified detectors^26^. However, despite a continuous development and strive towards faster sensor technology, all detection-based video configurations have a fundamentally limited frame rate due to the need to physically shift the generated photoelectrons^27^. This technological limitation has spurred the creation of alternative methods for videography that, instead of relying on rapid readout, rely entirely on high-speed *illumination*^28–30^. The acquisition rate of such illumination-based video systems are thus fundamentally limited by the pulse duration of the illumination and, with the advent of commercially available ultrafast pico- and femtosecond laser systems, has thereby enabled videography at THz video rates.

In this paper we present, to the best of our knowledge, the first demonstration of illumination-based schlieren videography, where the frame rate is governed by the pulse duration of the light source alone. The presented approach is based on an intensity modulation-scheme, which is a versatile optical method that is used for several different imaging applications, such as topography^31^, super-resolution microscopy^32^, schlieren^19,20,33,34^, interferometry^35,36^ and thermometry^37^. Here we combine its ability to provide schlieren images through phase-sensitive analysis^20^ with its ability to optically multiplex temporally separated image data^28,38–40^ into a powerful imaging concept. Past approaches for image multiplexing using this scheme have thus far been based on quantifying the amplitude of the intensity modulation; to gain schlieren data, we here demonstrate the ability to extract phase information from such multiplexed data. In the experiments we further verify the applicability of both coherent and incoherent light and demonstrate schlieren videography at acquisition speeds up to 1 Mfps, using a standard 50 fps sCMOS camera. We expect this imaging concept to open up for studies of transparent ultrafast, sub-nanosecond phenomena in the near future.

## Method

### Frequency recognition algorithm for multiple exposures

Frequency Recognition Algorithm for Multiple Exposures (FRAME) is an optical multiplexing technique that allows multiple image signals to be acquired within a single camera exposure (with a single detector), without having to read out each image individually^28^. This ability is enabled by marking each signal carrying light pulse with a unique spatial code, which is eventually used to identify and reconstruct the complete sequence of signals in the post-processing of the multiplexed camera acquisition. The character of the obtained sequence depends on how the light pulses are separated from each other in the optical part of the FRAME system. In the particular case of temporal separation the result is a video sequence^28^, but the separation could also be e.g. spatial^41^ or spectral^42,43^. For video sequences the minimum inter-frame time is only limited by the pulse duration, and the frame rate can therefore be significantly higher than that of the camera. To this date, frame rates of up to 5 THz have been achieved with the technique^28^.

**Figure 1:**
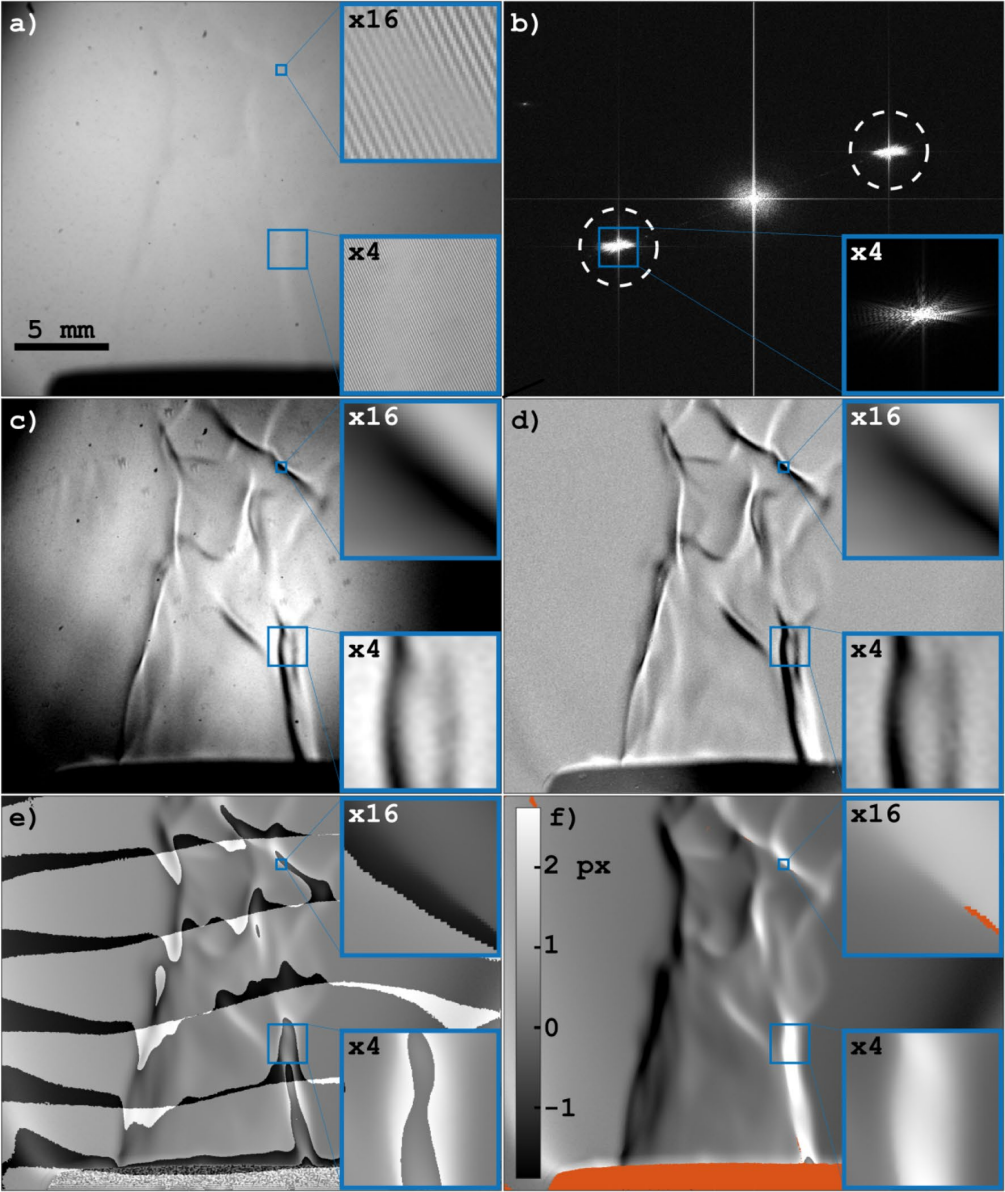
Illustration of the phase analysis process. (**a**) The image of the spatially modulated light pulse guided through a flame. The outlines of the flame are barely discernible, but leave a faint imprint in the modulated structure in the shape of a phase displacement. (**b**) The absolute value Fourier transform of the original image. The encircled Fourier clusters contain the information used to reconstruct the complex flame signal, carried by the light pulse. The size of the low-pass filter used for reconstruction is indicated by the circles. (**c**) The amplitude of the reconstructed signal. (**d**) The amplitude of the signal minus the amplitude of a blank recording. (**e**) The phase shift of the signal, i.e. the difference in phase between the signal and a blank recording. (**f**) The unwrapped phase shift of the signal. The grayscale bar gives the phase shift as displacement in pixels. The orange parts are areas where the amplitude of the signal was too low for proper determination of the phase. The zoomed in sections in (**a**) and (**c**)–(**f**) are of identical position. See also Supplementary video 1 for a comparison between pure trans-illumination imaging and schlieren based on modulated light.

**Figure 2:**
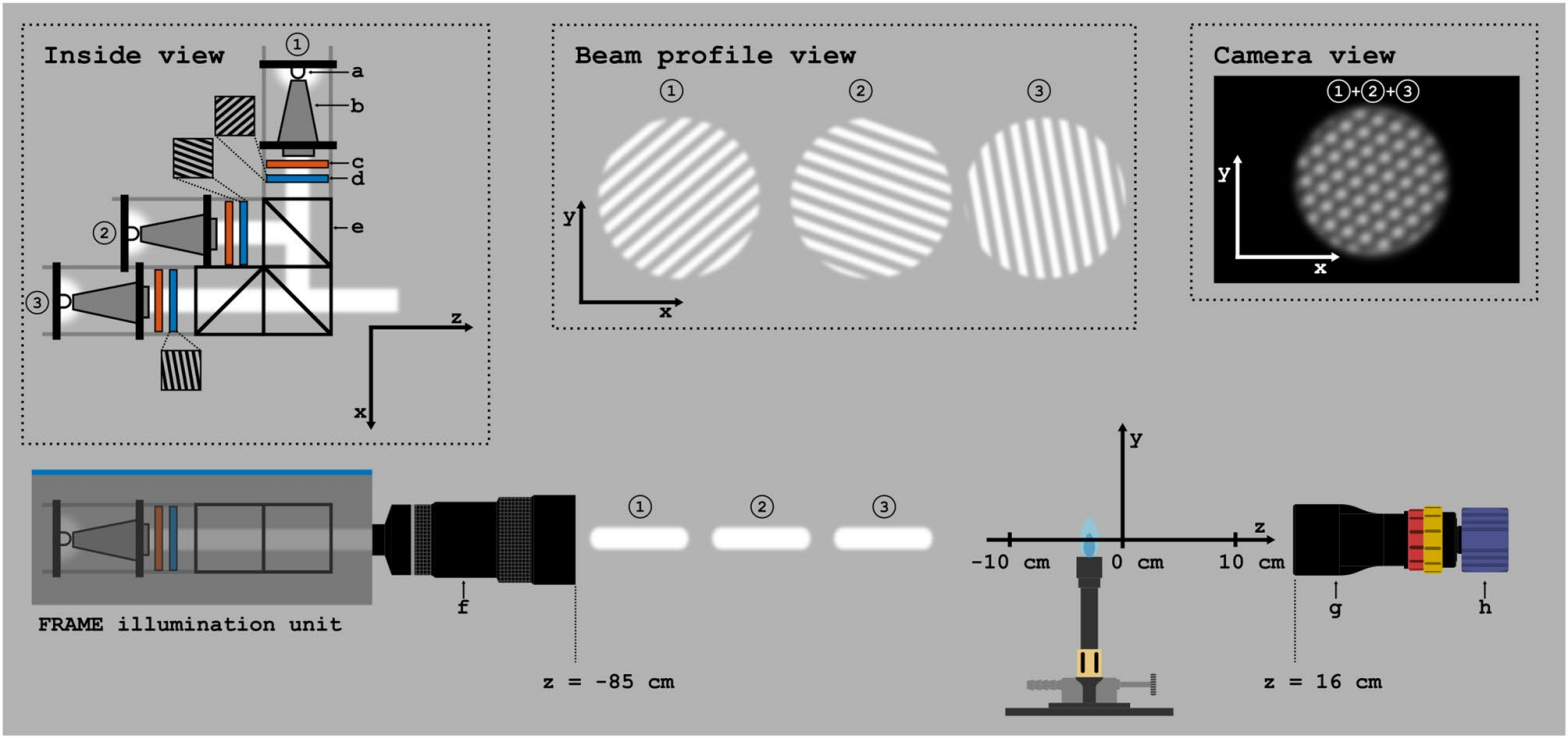
The experimental setup. A train of (up to) three modulated white light pulses illuminate a Bunsen burner flame and are recorded by a LaVision Imager M-lite 10-bit CMOS 5 MP camera (h). The pulse train is constructed by a FRAME illumination unit, where each optical arm consists of a fast LED (**a**), a tapered light pipe with 3x magnification (**b**), a diffusor (**c**), and a Ronchi grating (**d**). Recombination of pulses is performed by cube beam-splitters (**e**). The illumination unit is equipped with a Nikon 50–300 mm camera objectiv (**f**), while the camera (**h**) is equipped with an Edmund Optics Gold TL 0.5x telecentric objective, which has a fixed field-of-view of 26$\times$26 mm$^2$. (**g**). The Ronchi gratings are projected at the plane *z* = 0 cm. The modulation line widths and the shortness of the pulses are greatly exaggerated, while the distance from illumination unit to *z* = 0 cm is reduced for illustration purposes.

Figure 1 shows the signal extraction process of FRAME and how schlieren data can be measured using the method. Figure 1a shows a trans-illumination image of a premixed propane/air Bunsen burner flame that has been captured using the LED-based FRAME setup in Fig. 2. This image constitutes the *unprocessed* original data from which schlieren data will be extracted. The exposure time (10 μs) of this recording is too short for the flame luminescence to leave any noticeable imprints in the image and the faint outline of the flame is caused primarily by refraction of the light that transmits the flame. The main reason refraction becomes visible by eye (albeit only faintly) is because the incident light is guided through a transmission grating, which is subsequently imaged onto the detector. As seen in the zoomed in sections of Fig. 1a this spatially modulated illumination makes the image take on a striped appearance. It is this type of modulation that, not only makes refraction observable, but also makes optical multiplexing possible with FRAME. The reason multiplexing is possible is illustrated in Fig. 1b, where the image signal shows up not just in the centre of the Fourier domain, but also at positions corresponding to the angle and period of the modulation. In this context these signal copies are referred to as Fourier clusters. Even if the detector is subject to a series of signal-carrying light pulses, the respective Fourier clusters will have an insignificant overlap if the modulations of the pulses are sufficiently different from each other. This separation of Fourier clusters allow for their corresponding signals to be individually reconstructed, by applying a lock-in algorithm and a low-pass filter. The process is described in more detail in^28,38–43^. As more images are stored on the detector the distance between the Fourier clusters will unavoidably decrease, and eventually the size of the applied low-pass filter will have to be decreased to avoid cross-talk between signals. Hence, there is an inherent trade-off between the number of multiplexed images and the spatial resolution of the individual images associated with the technique. However, since for natural images most information is located close to the low frequency components in Fourier space, the losses can in practice be small, even for a large number of images^39^.

### Phase extraction methodology

When a spatially intensity modulated light field (such as that used in FRAME) propagates inside a transparent object, refraction leads to an observable displacement of the sinusoidal pattern. The amount of displacement at the location $\varvec{r}$ in the recorded image $I(\varvec{r})$ (see Fig. 1a), is determined in relation to a blank reference image $I_0(\varvec{r})$. The goal of synthetic schlieren imaging (which includes BOS) is to find the full 2D displacement field $\varvec{u}(\varvec{r})$ that transforms $I_0(\varvec{r})$ into $I(\varvec{r})$^20^, i.e. the $\varvec{u}(\varvec{r})$ that fulfills

$$\begin{aligned} I(\varvec{r}) = I_0(\varvec{r} + \varvec{u}(\varvec{r})). \end{aligned}$$

The entity $\varvec{u}(\varvec{r})$ is of interest, since it to first order is proportional to the refractive index gradients of the object under study. By applying Fourier domain filtering, centered on the modulation frequency in the recordings, the following signals are obtained:

1 $$\begin{aligned} \begin{aligned} s(\varvec{r})&= a(\varvec{r}) e^{i\varvec{k} (\varvec{r}-\varvec{u}(\varvec{r}))} \\ s_0(\varvec{r})&= a(\varvec{r}) e^{i\varvec{k} \varvec{r}}, \end{aligned} \end{aligned}$$

where **k** is the wave vector associated with the superimposed intensity modulation^44^. Taking the phase difference

2 $$\begin{aligned} \phi (\varvec{r}) = \text {arg}(s(\varvec{r})) - \text {arg}(s_0(\varvec{r})) = -\varvec{k} \varvec{u}(\varvec{r}) \end{aligned}$$

of the signals in Eq. 1 thus results in an equation that makes it possible to solve for the component of $\varvec{u}(\varvec{r})$ parallel to $\varvec{k}$. To obtain the full 2D displacement field two such equations, with $\varvec{k_1} \times \varvec{k_2} \ne 0$, are required.

Schlieren imaging, where the path-integrated variations of the refractive index is visualized in 2D, can thus be achieved by means of intensity-modulated illumination followed by a phase-sensitive analysis of the acquired data^21,45^. The phase-determination process is demonstrated in Fig. 1, where a single intensity-modulated light field has been used to probe a Bunsen burner flame. The Fourier transform of the acquired data shows the frequency component of the superimposed sine wave (Fig. 1b). By applying a 2D spatial lock-in analysis on the acquired image the complex signal $s(\varvec{r})$ (Eq. 1), containing both amplitude and phase information, carried by the modulated light pulse is retrieved. Past FRAME experiments have all been based on the extraction of the amplitude of the signal and performing such analysis on the data in Fig. 1a reveals otherwise barely discernible structures of the flame (Fig. 1c). Interfering background variations, rendered by an uneven illumination, can be mitigated through flat-field correction, i.e. subtraction of a blank recording (Fig. 1d). However, even though the amplitude image reveals structural information about the probed sample, it appears to primarily bring out finer details. For example, the unburnt- and the burnt regions appear to have similar contrast values in general, which is unexpected. We attribute these discrepancies to the fact that local variations in the amplitude of the signal are not directly related to the light displacement, i.e. schlieren data. However, as described above, the phase of the signal, rather than the amplitude, is the entity of interest for schlieren multiplexing as it carries information that can be transferred into the degree of light displacement. This transfer is achieved by calculating difference between the phase of the signal (i.e. the accumulated phase) and that of a blank recording (Eq. 2). Unfortunately the solution for the accumulated phase has many possible values (multiples of $2\pi$) and the outcome of the phase analysis (Fig. 1e) often needs to undergo an unwrapping procedure, where all appropriate multiples of $2\pi$ are considered and discontinuities (so-called $2\pi$-jumps) are minimized^46,47^. By properly extracting and unwrapping the accumulated phase across the FOV schlieren data is obtained, in which also smooth spatial structures in the reaction zone are visible (Fig. 1f).

### Experimental setup

To investigate the feasibility of combining FRAME image multiplexing and schlieren imaging, a LaVision FRAME illumination unit was used^38^. Apart from this unit, the setup consists of a 50 fps 10-bit 5 MP sCMOS camera (LaVision, Imager M-lite) equipped with a telecentric objective lens (Edmund Optics, Gold TL 0.5x) and a Bunsen burner that can be translated along the optical axis. The general layout of the setup is sketched in the lower part of Fig. 2. The modulated light pulses are supplied by the FRAME illumination unit, whose optical layout is shown in the upper left corner (“Inside view”). It consists of three optical arms, which are combined using beam splitters. At the start of each arm sits a Ce:YAG white light LED (Cree XHP35) with a 10 to 90% rise time of 205 ns, a 100 to 10% fall time of 500 ns and temporal jitter FWHM of 35 ns. The LEDs can be controlled individually, making it possible to set the duration of each pulse as low as 0.2 μs without restrictions in the inter-frame time. Ronchi gratings (50 lp/mm) are used in each optical arm to imprint a spatial modulation onto the intensity profile of the light pulses (“Beam profile view”). When observed through the camera, the grating period corresponds to a modulation period of 3.4 pixels per line pair. Once combined, the light pulses pass through a Nikon 50-300 mm camera objective, which images the gratings at the image plane (*z* = 0 cm). The temperature gradients in the Bunsen flame refract the light, thus inducing phase-shifts in the superimposed spatial modulations, which are imaged on the detector within the same acquisition (“Camera view”). The FOV of the proof-of-concept system is currently set to 26$\times$26 mm$^2$, restricted by the telecentric lens. Note, however, that schlieren based on intensity-modulation can be used to visualize larger objects^34^, as demonstrated in Fig. S4, where the FOV is 110$\times$110 mm$^2$ and Supplementary video 2 and 3.

## Results

### The effect of object position on sensitivity

**Figure 3:**
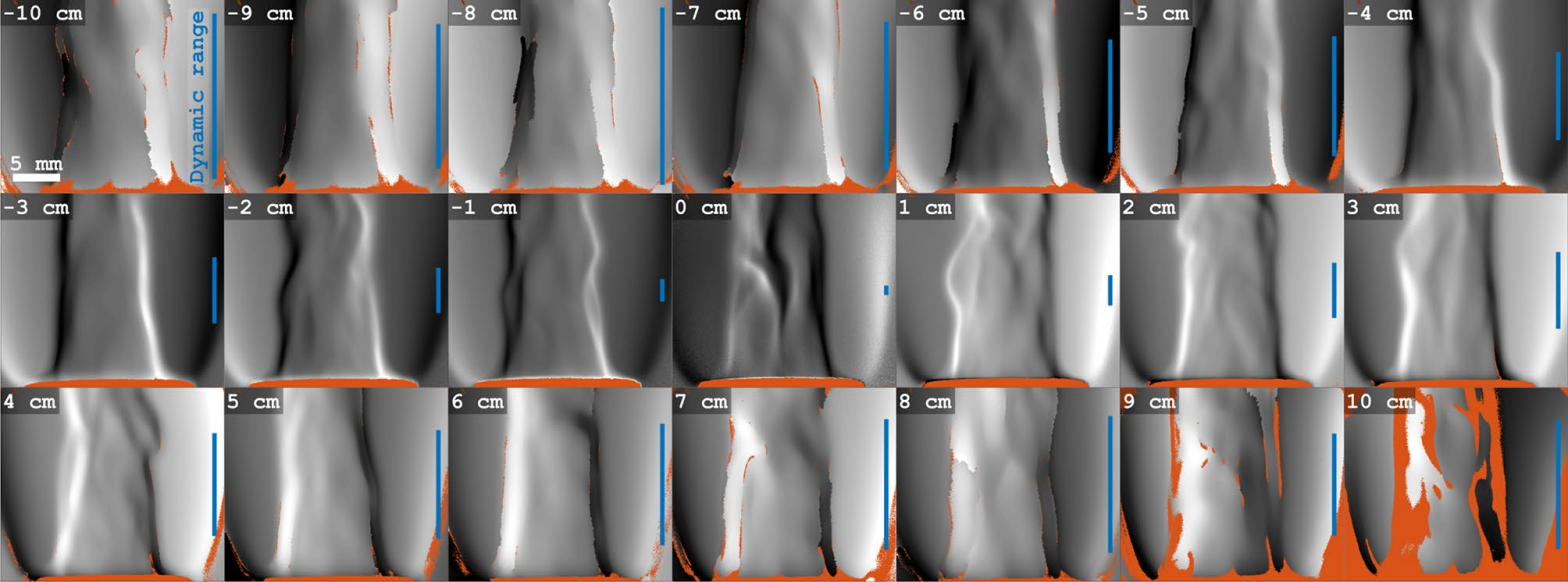
Phase shift images for varying target positions. The images have been acquired and reconstructed according to the FRAME method, followed by phase unwrapping. Negative distances correspond to positions closer to the illumination. Orange areas indicate where the amplitudes were too low for accurate phase determination. The blue lines show the relative dynamic range of respective image, i.e. the difference between the maximum and the minimum phase value.

One of the main drawbacks with a BOS system is that the image plane of the textured background (or modulated illumination) must not coincide with the object plane of the sample under study^4,48^. As the analysis of the displacement relies on visualizing fine distortions in the background pattern, the object inevitably needs to be placed out-of-focus from the cameras perspective. This requirement leads to a trade-off between sensitivity and spatial resolution; a large distance between object- and image plane yields greater displacements but allows only for visualization of the main features of the sample. The opposite case, where the object- and the image plane coincide, reveals finer structural details but the sensitivity in quantifying the displacement may be significantly reduced (especially for flatter objects). Our setup is a variation of a traditional BOS arrangement, meaning that it also is subject to this trade-off. To investigate the relation between spatial resolution and measurement sensitivity inherent in our system, 21 schlieren images of a Bunsen burner flame were acquired at different object positions, see Fig. 3.

The results in Fig. 3 show the expected trend; a greater distance between the image plane and the position of the object yields a more pronounced phase shift of the incident intensity modulation. When the burner is placed at *z* = 0 cm, i.e. at the image plane, the dynamic range of the phase shift is reduced by a factor of $\sim$17, compared to $z = \pm 10$ cm. At the same time, positioning the object too far away yields a reduction in the amplitude of the superimposed intensity modulation that, in turn, makes accurate phase determination challenging. Regions where the signal amplitude is less than the average noise level—and thus deemed unfit for accurate phase reconstruction—are indicated by the orange areas, which can be seen to increase in size as the burner is placed farther from the image plane. These results are in consonance with established BOS theory, which states that the sensitivity of the system is approximately proportional to the distance between the object of study and the reference background^49^. As for all BOS systems, the trade-off between sensitivity and the distance between the object and the focal plane must thus be considered. For the following results, the Bunsen burner was placed at $z=-3$ cm.

### The effect of low-pass filter size

Another factor that influences the spatial resolution in the schlieren images is the characteristics of the low-pass filter used to isolate and extract the multiplexed images in the data post-processing. In general, a wider filter permits higher spatial frequencies but increases the risk of cross-talk between neighboring images (in the Fourier domain). However, a too wide filter would not necessarily amount in more structural details, as explained by the Nyqvist-Shannon sampling theorem^50,51^. This theorem states that a single intensity modulated field with a carrier frequency of $\nu _{mod}$ can, at best, be used to extract spatial variations corresponding to $\nu _{mod}/2$.

**Figure 4:**
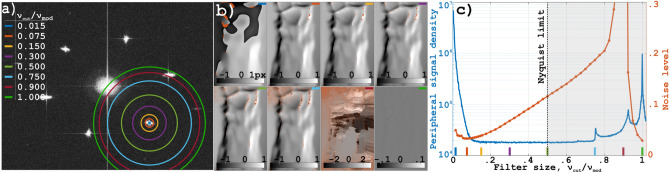
The effect of filter size on image quality. (**a**) The absolute value of the Fourier transform of the original image. The rings indicate the sizes of the filters used to reconstruct the signal in the Fourier cluster they are centered on. In the legend the filter specific ratio between its cut-off frequency and the modulation frequency is given. (**b**) Light displacement in pixels for different filter sizes. The colors in the upper right corners show the filter size, according to the legend in (**a**). (**c**) The measurement’s peripheral signal density, defined as the total signal at the (one pixel wide) filter edge divided by the area of the filter edge (blue line, left y-axis), and the noise level, defined as the average standard deviation of 10 000 random small areas ($10\times 10$ px), in images as those in (**b**), for 49 different filter sizes (orange, right y-axis).

To investigate how the filter size influences the quality of the extracted multiplexed schlieren images,a set of 49 low-pass filters with different bandwidths were used to extract the bottom right sub-image of Fig. 6. The bandwidth of each filter is characterized by a cut-off frequency $\nu _{cut}$, which can be expressed as a fraction of the modulation frequency $\nu _{mod}$ (see Fig. 4a).The schlieren images in Fig. 4b show that using a filter with $\nu _{cut}=0.015 \nu _{mod}$ yields an unsatisfactory result, having both a poor spatial resolution and an unsuccessful unwrapping. However, already for $\nu _{cut} \ge 0.075\nu _{mod}$ the unwrapping becomes stable and the signal appears to be fully captured; increasing $\nu _{cut}$ further does not reveal any new structures in this particular object, but instead the noise level increases. This effect is quantitatively shown in the graph in Fig. 4c, where the orange line represents the noise level in the extracted images (measured by taking the average standard deviation of 10 000 randomly selected areas of 10x10 pixels) as a function of $\nu _{cut}$. The graph shows that the noise level reaches a minimum when applying a filter with a $\nu _{cut} \approx 0.075 \nu _{mod}$. For lower values of $\nu _{cut}$, the noise increases due to unsuccessful phase unwrapping and hence the introduction of artificial sharp edges in the image. For higher values of $\nu _{cut}$, cross-talk with neighboring images are partly responsible for the increase in the noise level, yet we attribute this increase primarily to the fact that image signals, in general, drop as $1/\nu$ and a larger filter bandwidth would therefore mostly add noise. Plotted in Fig. 4c is also the signal density at different distances from the Fourier cluster, shown as a blue line. Close to the center of the Fourier cluster the signal density is high, but decreases rapidly as the distance to the centre increases. From $\nu _{cut}\approx 0.15 \nu _{mod}$ to $\nu _{cut} \approx 0.75\nu _{mod}$ there is a plateau of low signal density, which indicates that the contribution in this area is primarily the constant background noise. Beyond $\nu _{cut} \approx 0.75 \nu _{mod}$ the signal density increases as a result of other Fourier clusters being included, i.e. cross-talk. These results suggest that a bandwidth of $0.075 \nu _{mod}$ is sufficient for the current measurements.

### The effect of modulation period on sensitivity

**Figure 5:**
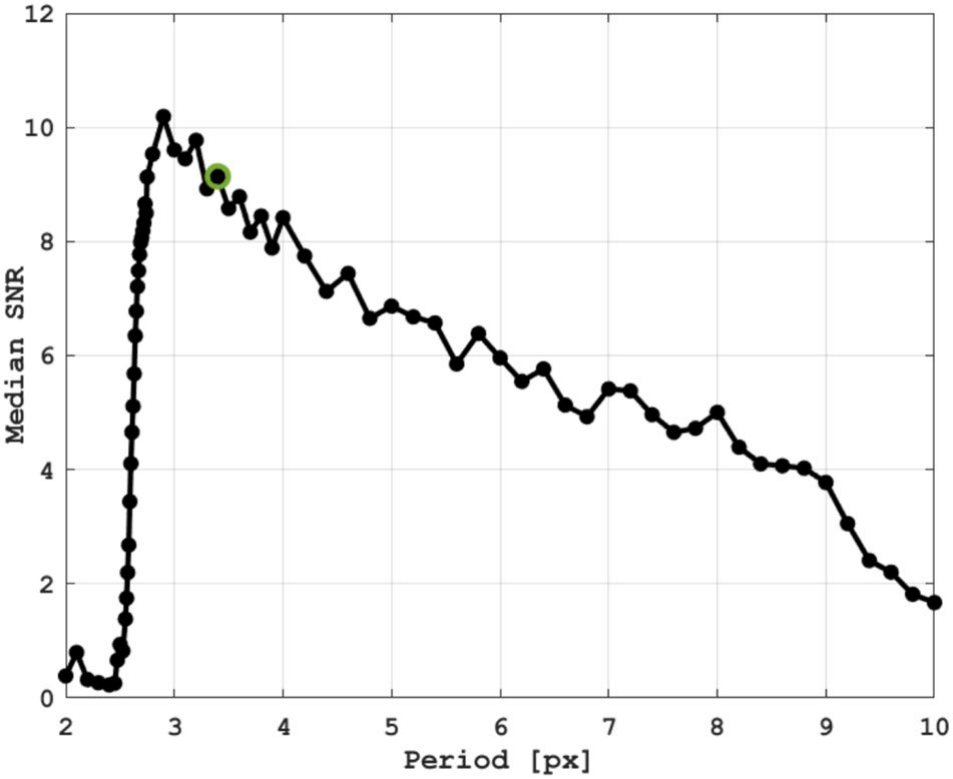
Modelled signal-to-noise ratio as function of modulation period. Each data point is a modelled median signal-to-noise ratio for a specified modulation period. The model is described in the Supplementary information. The highlighted data point (green circle) corresponds to the modulation period of the experimental data. The analysis shows that the measurement sensitivity is poor at low- and high modulation frequencies (near the Nyquist limit) and that a periodicity of  3 pixels yields highest sensitivity.

A model that generates synthetic data, closely resembling the experimental data, was developed to study how the period (1/$\nu _{mod}$) of the intensity modulations affects the signal-to-noise ratio (SNR) of the detected phase shift. The synthetic data contains a central region where a variable phase is added, yielding a displacement of the modulations in this region (see Fig.S1–S3). After applying the phase analysis process, described above, the SNR is determined as the measured difference in phase between the two regions divided by the average standard deviation over the entire image. This analysis is repeated as the displacement is scanned from 0.01 pixels to the modulation period, which, in turn, is varied from 2 to 10 pixels. The sensitivity, as a function of modulation frequency, is then determined by calculating the median SNR over each scan (see Fig. S2), yielding the trend shown in Fig. 5.From this trend it can be concluded that the sensitivity of schlieren signals based on intensity-modulation depends highly on the modulation period, with a maximum at  3 pixels. The experimental modulation period (3.4 pixels) is thus close to the optimum period. According to these results, sub-pixel displacements—down to 0.02 pixels (SNR $\approx$ 2)—can be achieved under the current experimental conditions. See Supplementary information for additional information and example images.

### Single-shot optically multiplexed schlieren videography

**Figure 6:**
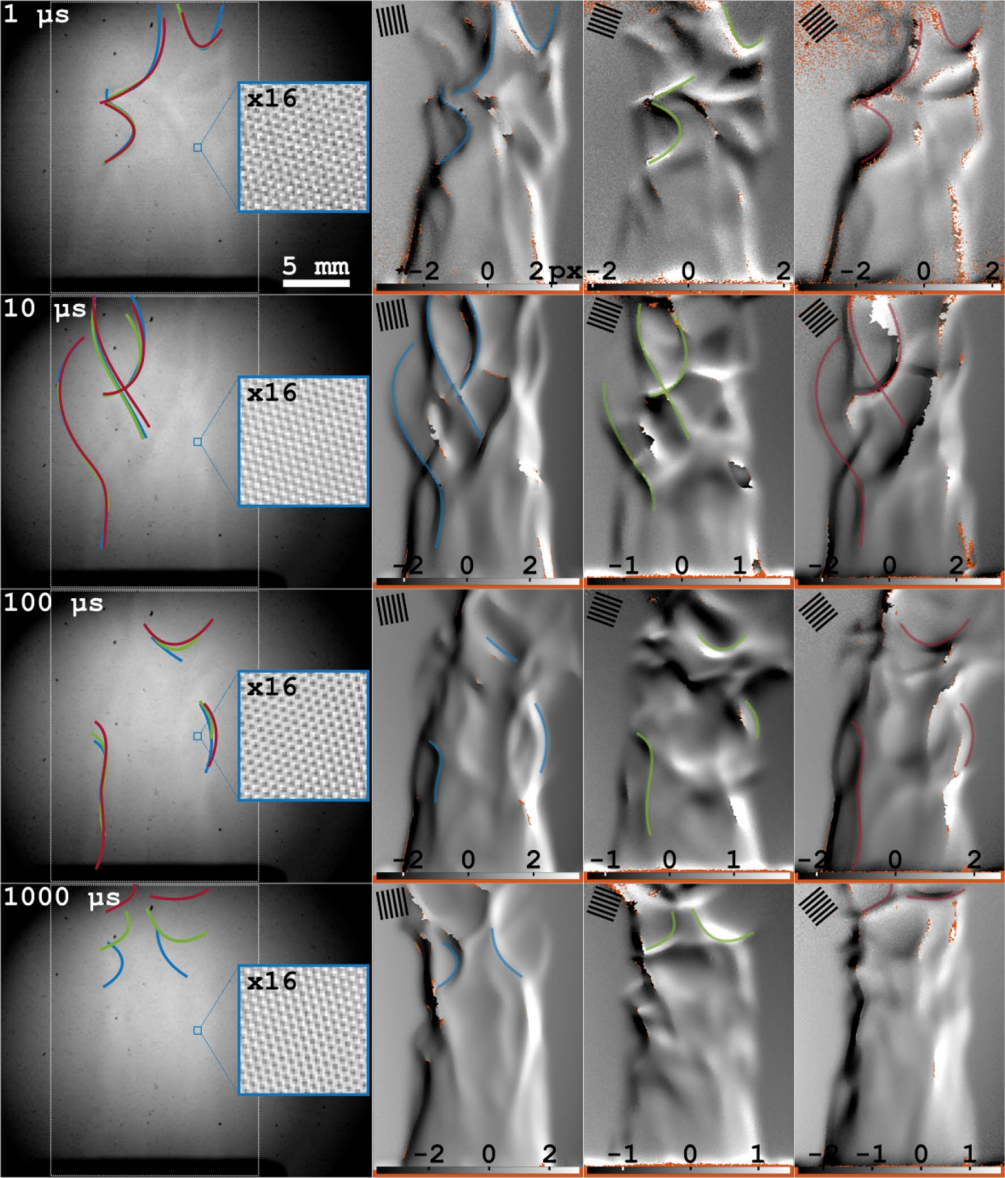
Video sequences of flame induced light displacement. The left column show the original image for each sequence. The blue, green and red lines highlight structures that are considered identical in image 1, 2 and 3, respectively. These colored lines are present both in the original image and in the image they originate from. The inter-frame time of each sequence is shown in the upper left corner of the original image. The orientations of the spatial modulations, used for acquisition, are shown for respective image.

The purpose of FRAME is to simultaneously store more than one signal (image) on a single detector—an ability enabled by means of optical multiplexing. To date, all experiments using FRAME have been based on quantifying the local amplitude of the superimposed intensity modulated pattern associated with each multiplexed image. This amplitude is either reduced by the sample through e.g. light extinction^38,39^ or is increased due to e.g. fluorescence^43^. In this study we investigate the possibility to quantify the local phase of a set of temporally distinct, yet multiplexed intensity modulated image signals, which ultimately would open up for ultrafast schlieren videography. To investigate this and determine whether refractive index gradients in transparent objects can be visualized by means of FRAME, 4 video sequences—each composed of 3 frames - with a respective inter-pulse time of 1, 10, 100 or 1000 μs of a Bunsen burner flame was captured (Fig. 6). To avoid temporal overlap between images in the two fastest sequences, the pulse duration was set to be equal to the inter-pulse time (1 and 10 μs). For the other two sequences the pulse durations were set to 30 μs, to avoid motion blur and overexposing the detector. These differences in exposure time (pulse duration) will influence the SNR, which inevitably becomes lower as the exposure time is reduced. This reduced SNR can be noticed both in the unprocessed camera recordings (see magnified regions) as well as in the extracted sequences.

The steepest temperature gradient in the Bunsen flame is at the reaction zone (flame front), which is the sub-millimeter boarder between the burnt and the unburnt air/propane mixture^52^. The majority of light refraction occurs in this interface and the schlieren images are therefore expected to primarily carry information about this region. The unprocessed image (as recorded by the camera) for each sequence is presented in Fig. 6, wherein a faint imprint of the wrinkled structure of the reaction zone can be discerned (likely as Moiré patterns). This imprint diminishes as the $\Delta$t between the multiplexed frames increases because of the motion of the flame, which will smooth out structural details. By applying the spatial lock-in analysis on the acquired image and determining the local phase shifts, the progression of the complexly wrinkled flame front is revealed with high contrast. At short $\Delta$t the wrinkling of the flame is frozen in time, as illustrated by the spatially overlapping highlighted structures (colored lines), while the corresponding structures do not overlap in space for sequences with a $\Delta$t of 100 and 1000 μs. The extracted schlieren images show no signs of cross-talk, which would be manifested as intensity-modulated structures. These results and findings demonstrate that optical multiplexing of temporally separated images can indeed be united with schlieren sensitive imaging.

One drawback with the image-coding methodology associated with FRAME multiplexing is that it is one-dimensional, i.e. each light pulse is intensity modulated by a single spatial frequency $\nu _{mod}$, which does not permit the estimation of the displacement vector component parallel to the stripes^20^. Since the image-coding of FRAME relies on the multiplexed images having different modulated patterns, this inability poses a problem; the individual frames will have uneven capabilities in resolving structural details depending on the orientation of the superimposed intensity modulation. This difference in resolving capabilities can be seen in the image sequence with a $\Delta$t of 1 μs, where, despite the inter-frame time being sufficiently short to temporally freeze the rapidly evolving flame structures, certain structures in which the flame fronts appear different can be identified. Such structural differences can be mitigated by reducing the angle between the intensity modulations in the illumination, yet with an increased risk of image cross-talk.

### Schlieren videography with full vector displacement

A more complete solution to the problem of measuring spatial variations in refractive index orthogonal to the modulation was presented in 2018 by Wildeman^20^; by using a checkered background pattern, i.e. composed of multiplicatively combined vertical and horizontal binary lines, the phase shift (and hence the light displacement) can be estimated in both the x- and y-direction. To investigate the feasibility of combining this approach for full vector displacement with temporal image multiplexing, a laser-based FRAME setup was used, capable of producing four intensity-modulated laser pulses with an intra-pair time separation of 6 μs. Using this setup, the Bunsen flame could thus be illuminated with a set of two temporally separated intensity modulated pairs of laser pulses with orthogonal modulations.

**Figure 7:**
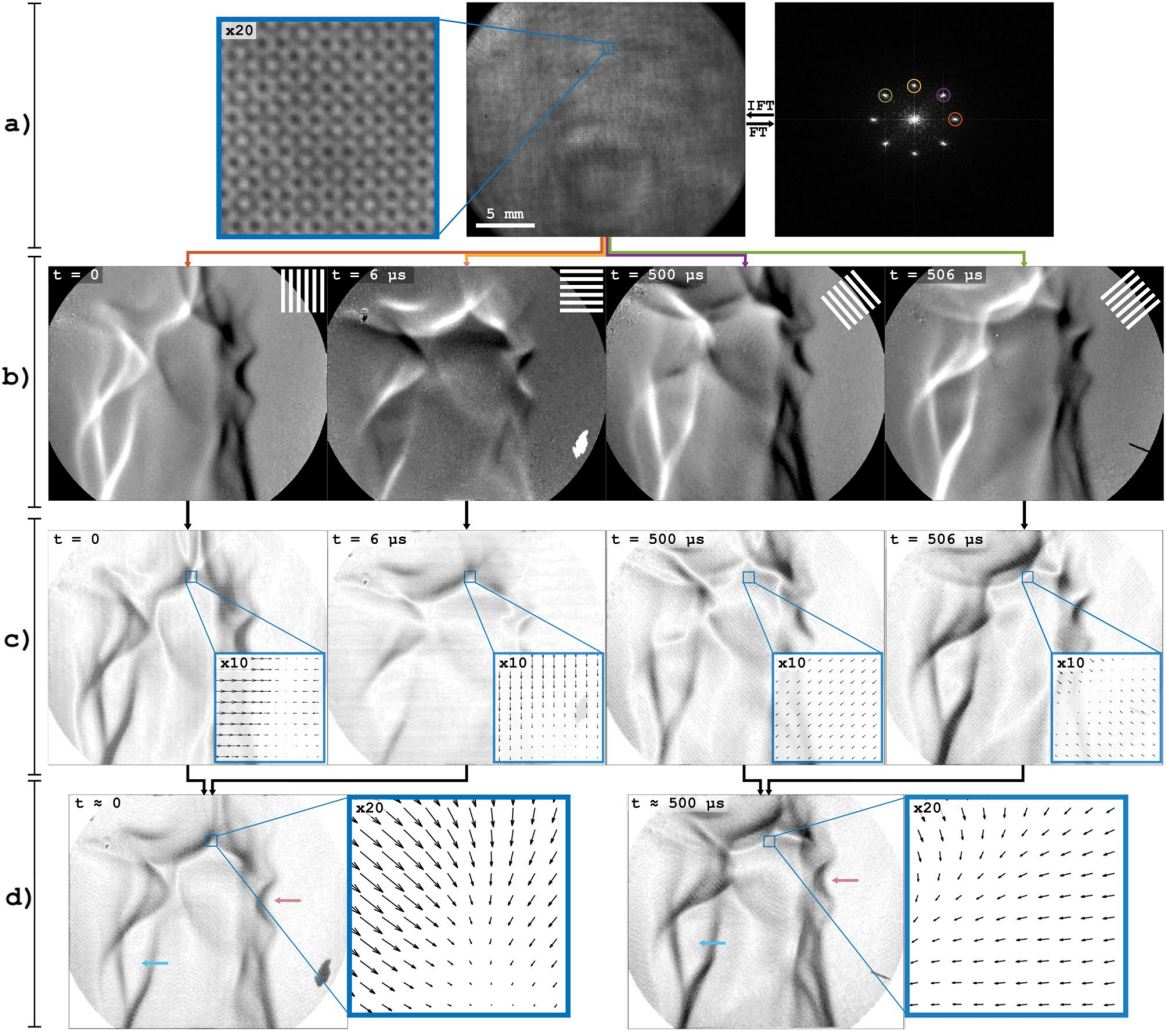
Videography using orthogonal modulations. (**a**) The original image and the absolute value of its Fourier transform. (**b**) Images showing the magnitude of the flame induced displacement. (**c**) Magnitude and direction of the displacement, shown through arrow plots. (**d**) The displacement vectors of both signal pairs, i.e. signals recorded with the minimum possible temporal separation of 6 μs, are added to get the full 2D vector displacement at both recording instances. The red and blue arrows point at (what is considered) identical structures in the flame. The zoomed in sections are of identical position.

Figure 7a displays an image of the Bunsen flame captured using the laser-based FRAME setup. The images in Fig. 7b show the magnitude of the light displacement induced by the flame at unique times and in unique directions. The direction and magnitude of the displacements are shown as arrow plots in Fig. 7c. In Fig. 7d the arrow plots associated with (approximately) the same time have been combined by adding the components of the vectors, yielding two arrow plots showing both direction and magnitude of the full 2D vector displacement induced by the flame at two different occasions. These results illustrate the possibility of using image multiplexing in order to, in 2D, track complex structural details of transparent objects that evolve at high speeds.

## Discussion

The family of schlieren-like techniques is a powerful set of tools that can be used to visualize and even measure refractive index gradients in transparent media. However, no schlieren technique has previously been demonstrated in combination with videography based on image multiplexing. Schlieren videography has thus been confined to acquisition speeds dictated by camera frame rates, limiting what phenomena can be studied accordingly. In this study we exploit intensity-modulated illumination in order to (1) qualitatively visualize the refractive index gradients of the object and (2) multiplex a set of temporally separated images from which schlieren data can be extracted. This FRAME-based schlieren technique resembles synthetic schlieren and FCD, but differs in one crucial aspect; instead of a static backdrop it utilizes a dynamic background, in the form of a burst of modulated light pulses. The implication of this is that the frame rate is not set by the camera, but by how short light pulses the illumination source can deliver. By instead relying on rapid illumination, slow yet high-resolution sensors can be used for fast videography. Further, by using both LED and laser sources to produce the modulated light burst, we show that the functionality of the technique is independent on whether or not the light source is coherent.

A drawback with the FRAME technique is that the number of frames in the sequence is limited. However, it has previously been demonstrated that hundreds of signals can be multiplexed in this way^39^, and our results (Fig. 4b) suggest that it could be sufficient with a Fourier space area as small as $100 \times 100$ pixels ($\nu _{cut} = 0.075 \nu _{mod}$ corresponds to a filter radius of $\sim$50 pixels) to capture all relevant signal, while still avoiding cross-talk between signals. Given these conditions, approximately 30 image signals could be multiplexed on a 4 Mp sensor using 4 px/lp modulations (corresponding to Fourier clusters 1000 pixels from the centre, so that $n \approx 1000 \pi / 100 \approx 30$). However, perhaps the main challenge associated with increasing the number of multiplexed signals by a certain factor *x* is that the level of the individual signals will have to decrease by the same factor to avoid overexposing the detector. The noise that the signals are competing against is however global, and will therefore not decrease, meaning that the SNRs will decrease by the factor *x*. Choosing the smallest possible filter size, to reject as much noise as possible, while still capturing the full signal, will thus be crucial for long video sequences. For more details on the challenges—both concerning the illumination as well as the analysis—with multiplexing long video sequences, see Ref.^39^.

In previous FRAME studies, the amplitude has been the sought part of the reconstructed signals. For most applications, e.g. those based on absorption, extinction and fluorescence, the amplitude is the important aspect of the signal, but when studying transparent objects it is not ideal, even though the flame refractive index gradients can be seen to induce amplitude variation (Fig. 1c,d). This is because the relation between object’s refractive index and the observed amplitude is dependent on how the optical signal is digitized, and hence not easily interpreted. The phase shift, however, is directly related to the displacement of light, which is a result of the encountered refractive index gradients. It can thus reveal information about the structure of the object under study. Here we show that the phase can be retrieved after multiplexing 4 images on the same detector.

Based on the results in this study it is evident that FRAME can be used to generate schlieren images of dynamic transparent events, revealing how the refractive index within an object changes. To the best of the authors knowledge this is the first time multiplexed illumination-based schlieren videography has been demonstrated. Since, for these techniques, the achievable frame rate is solely limited by how short light pulses one can, produce, this paves the way for future ultra-high speed schlieren videography.

## Supplementary Information

Supplementary Information 1.

Supplementary Information 2.

Supplementary Information 3.

Supplementary Information 4.

## Data Availability

Data underlying the results presented in this paper are available from the corresponding author upon request.
