# Supplementary material for: High-speed videography of transparent media using illumination-based multiplexed schlieren
Source: Sci Rep. 2022 Nov 8;12:19018. doi: 10.1038/s41598-022-23198-6 (PMC9643512; doi:10.1038/s41598-022-23198-6)
Supplement: Supplementary file 1 — Supplementary Information 1. [file 41598_2022_23198_MOESM1_ESM.pdf]

SUPPLEMENTARY INFORMATION

Modelling phase extraction sensitivity. As a light field propagates through a transparent object with spatial variations in refractive index it becomes distorted. Schlieren imaging based on intensity-modulated light utilize this effect and by analyzing the phase shift the distortions give rise to, the otherwise transparent object becomes visible. The sensitivity in detecting spatial variations in refractive index thus depends on the ability in detecting a phase shift. Experimentally, this sensitivity is challenging to investigate, as it would require a well-defined object with a variable refractive index. Instead, we perform computer simulations, where the phase shift can be controlled and adjusted with sub-pixel accuracy.

The simulations are based on 64×64 pixel area, see Fig. S1a)-b). To replicate the experimental conditions in terms of the intensity level, three parameters - intensity offset, signal level and noise - were adjusted until the histogram of the synthetic data agreed with that of the experimental data, Fig. S1c).

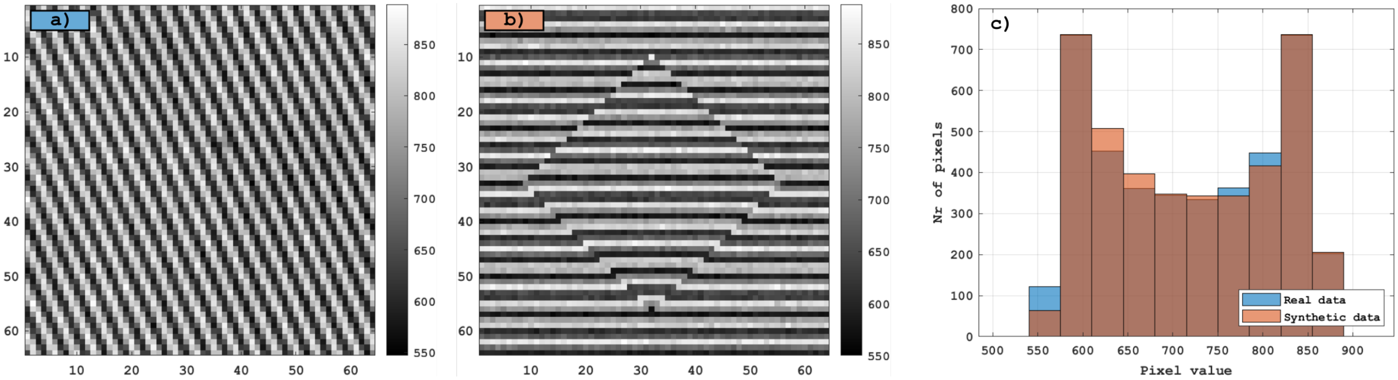

Fig. S1. Comparison between real and synthetic data. a) A 64×64 section of unprocessed real (experimental) data. **b)** Synthetic data with identical overall size and modulation period, but with a 32×32 pixel central section with added phase, in this case corresponding to a modulation displacement of exactly 1 pixel. **c)** A histogram showing the pixel value distribution for the real and synthetic data, respectively. The close agreement indicates that the selected values for the frequency and added noise are correct.

To determine the sensitivity of the phase detection, the phase of the intensity modulation in a sub-region (32×32 pixels) of the synthetic data was gradually altered and then analyzed using the spatial lock-in algorithm and unwrapping procedure described above. An overview of the results of the simulations are displayed in Fig. S2b), where the applied modulation frequency in Fig. S2a) is matched with that of the experiment ($\nu_{mod} = 1/3.4$ pixels^{-1}). Given the current experimental conditions, the simulations show that the phase-shifted sub-region becomes detectable with the spatial lock-in algorithm and phase unwrapping procedure already at a phase-shift corresponding to 0.02 pixels, i.e. sub-pixel sensitivity.

The sensitivity can be quantified in greater detail by estimating the SNR as a function of modulation displacement, where the signal is measured as the difference in phase between the phase-shifted sub-region and the surrounding region, and the noise as the mean standard deviation of the two regions (*after* post-processing the data). Such an analysis is displayed in Fig. S3, where also five additional modulation periods are included (see also Fig. 5). The general trend found here is that for sufficiently large modulation periods ($\gtrsim$2.7 pixels) the SNR increases rapidly as the modulation displacement increases to approximately 0.25 pixels. From 0.25 pixels worth of displacement the SNR increases more slowly and then dips, as the displacement approaches half the modulation period. For symmetry reasons the SNR curves become mirrored around a displacement corresponding to half the modulation period. The analysis shows that high modulation frequencies - modulation periods below 2.7 pixels - yield low SNR values across the entire scan and are thus unsuitable for schlieren imaging. The relation between median SNR and modulation displacement is displayed in Fig. 5.

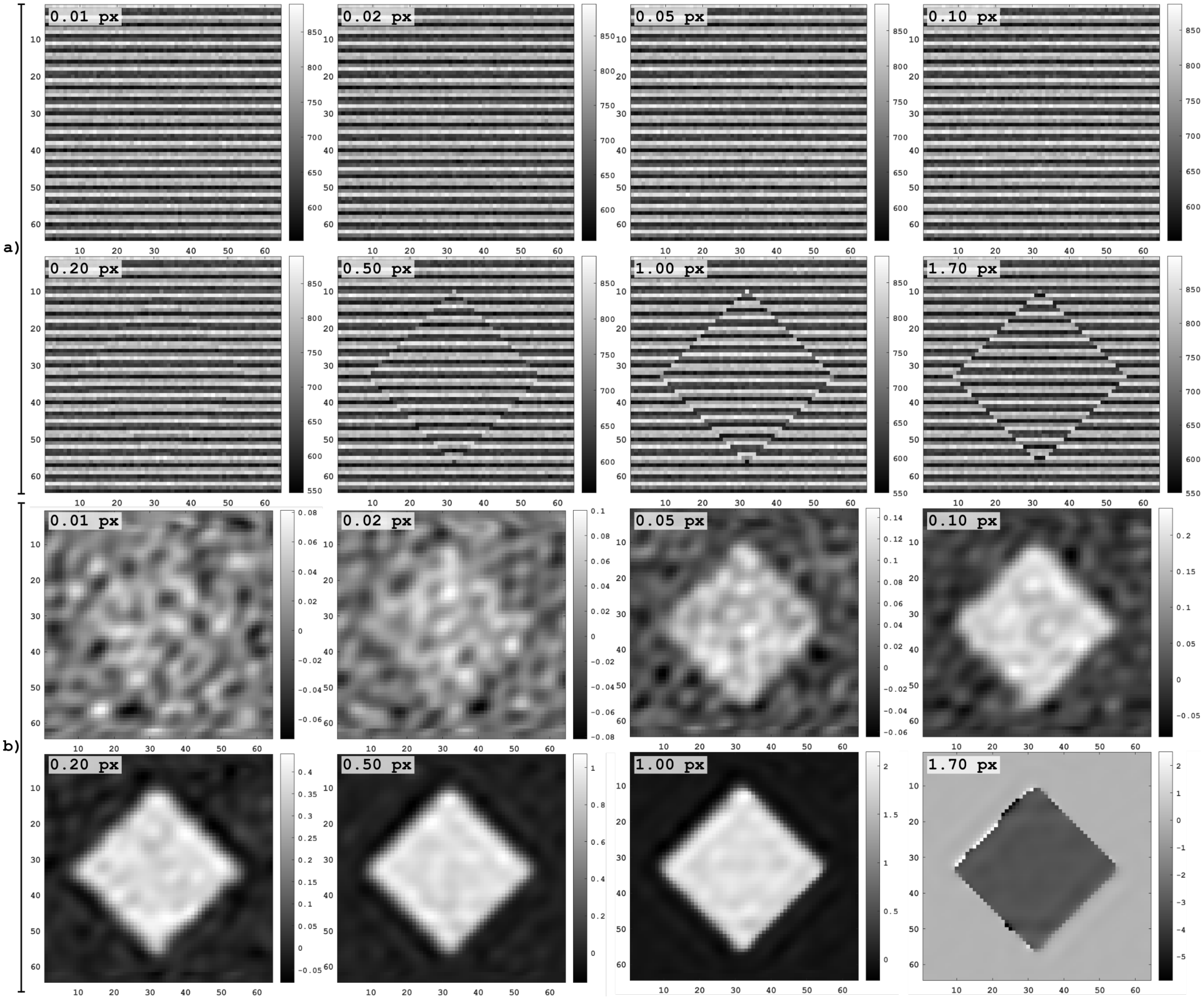

Fig. S2. Original synthetic data and extracted phase maps. a) Each original image is 64×64 pixels, have a modulation period of 3.4 pixels (equal to the experimental data) and a 32×32 central region where the phase is shifted. The displacement (in pixels) is shown in the upper left corner of each image. **b)** Phase maps extracted - using the exact same methodology as for experimental data - from the corresponding original image.

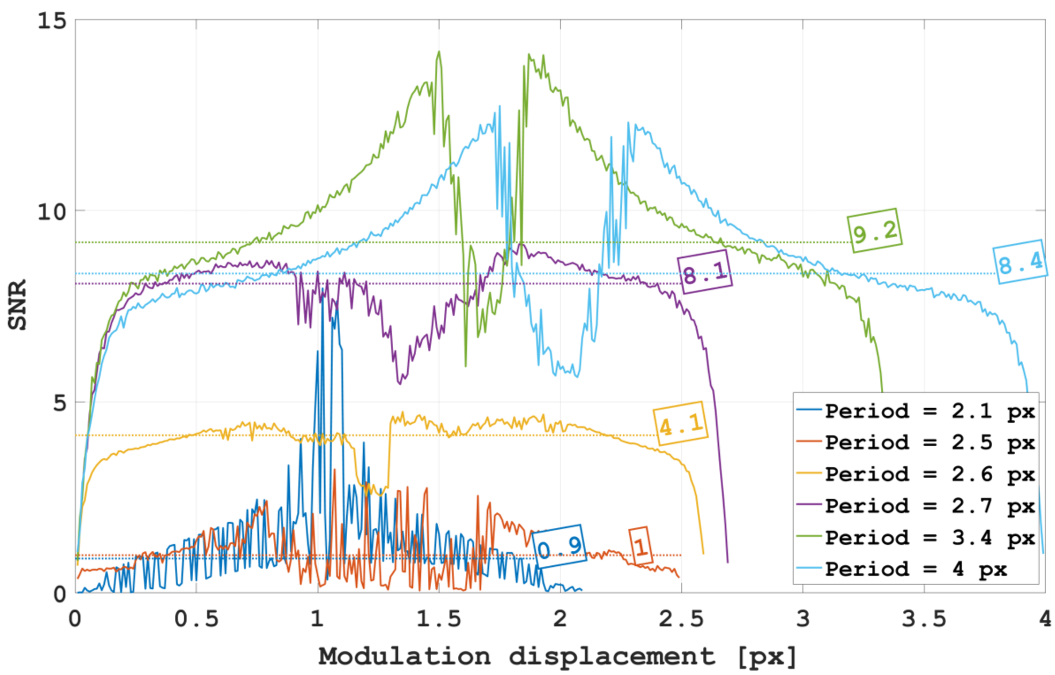

Fig. S3. Modelled signal-to-noise ratios as function of modulation displacement. Each line corresponds to the modelled SNR as function of modulation displacement for a specific modulation period. The dotted lines and boxed values show the median SNR for respective modulation period.

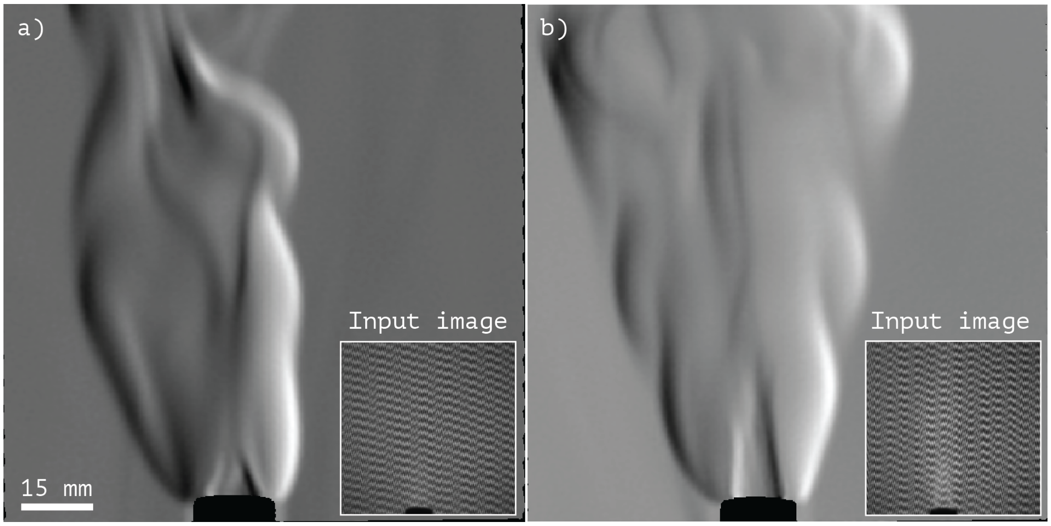

Fig. S4. Bunsen flame visualized at a large FOV. In this experiment, the Bunsen burner was placed in front of a computer screen upon which a modulated pattern was displayed, allowing the flame to be captured at a large FOV (110×110 mm^2.). For comparison, the burner was operated at either a low- (a) or higher flow speed (b). See also Supplementary video 2 and 3.

Supplementary Videos

Supplementary Video 1. Comparison between contrast achieved using either trans-illumination videography or intensity-modulated schlieren. The videos demonstrate how refraction caused by the temperature gradients yield negligible contrast when observing the transmitted light (left video), whereas such gradients become visible when extracting the phase shift of the superimposed intensity modulation (right video).

Supplementary Video 2. Intensity-modulated schlieren of a premixed propane/air Bunsen flame operated at a reduced flow rate, captured at a large field-of-view (110$\times$110 mm^2) using either trans-illumination (left video) or intensity-modulated schlieren (right video).

Supplementary Video 3. Intensity-modulated schlieren of a premixed propane/air Bunsen flame operated at an increased flow rate, captured at a large field-of-view (110$\times$110 mm^2) using either trans-illumination (left video) or intensity-modulated schlieren (right video).
