# Supplementary figures and images for: High-speed videography of transparent media using illumination-based multiplexed schlieren
Source: Sci Rep. 2022 Nov 8;12:19018. doi: 10.1038/s41598-022-23198-6 (PMC9643512; doi:10.1038/s41598-022-23198-6)

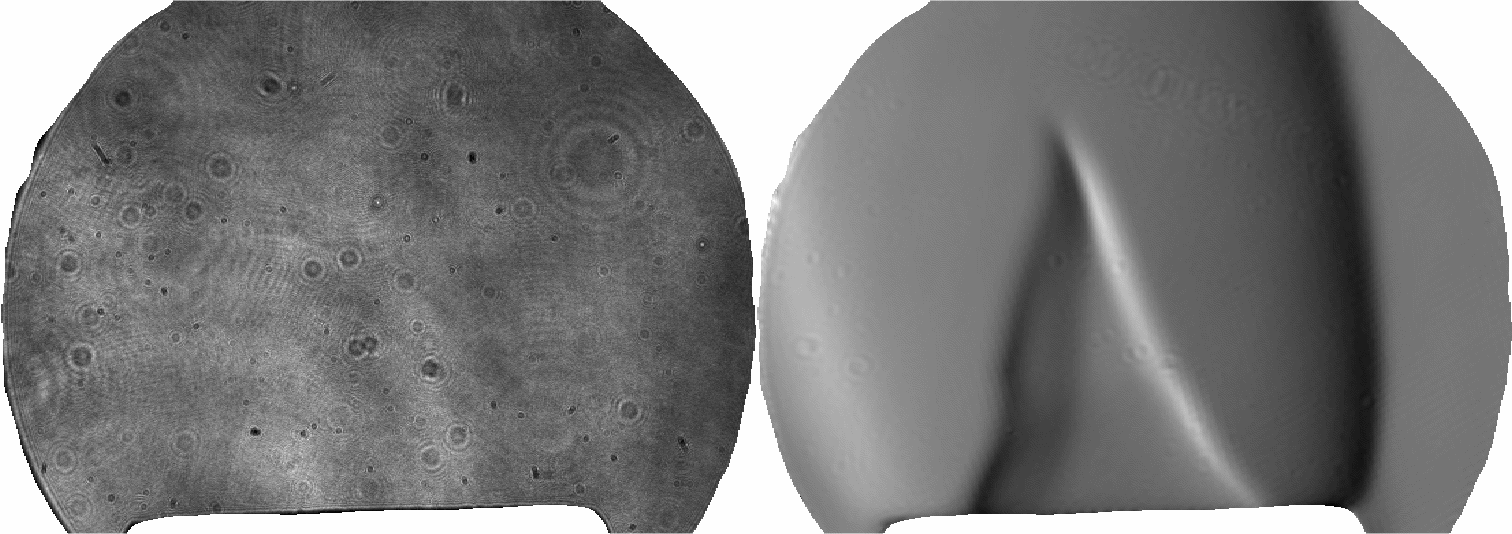

Supplement: Supplementary file 2 — Supplementary Information 2. [file 41598_2022_23198_MOESM2_ESM.gif]

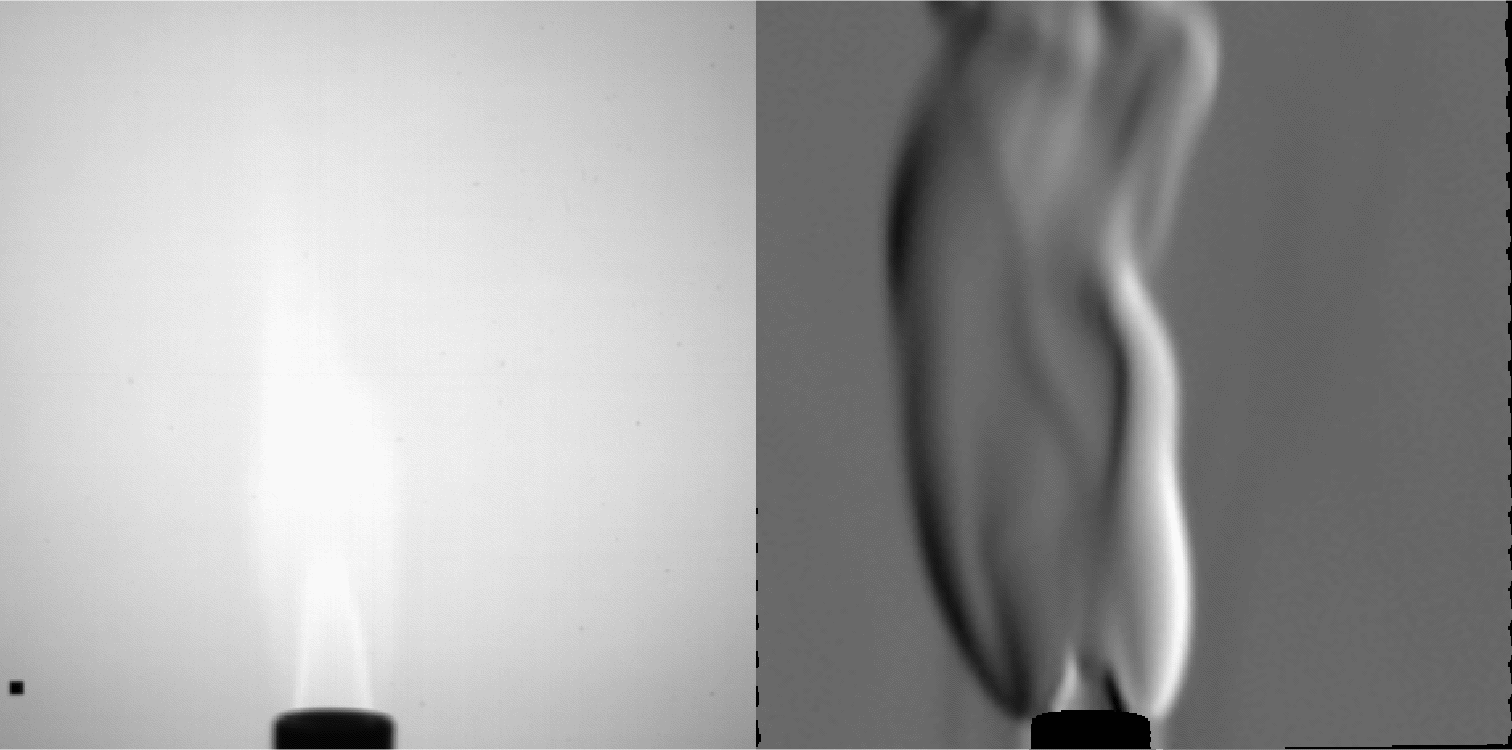

Supplement: Supplementary file 3 — Supplementary Information 3. [file 41598_2022_23198_MOESM3_ESM.gif]

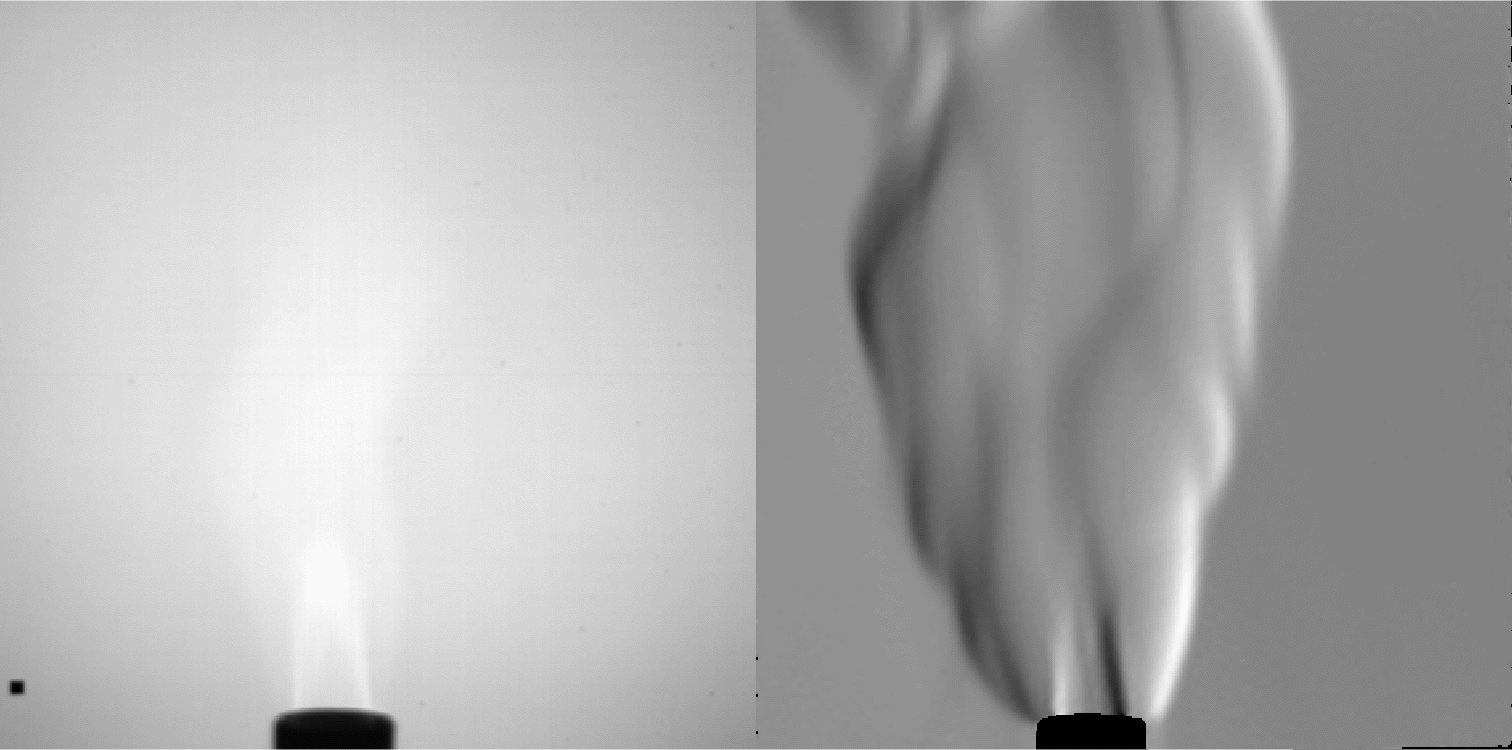

Supplement: Supplementary file 4 — Supplementary Information 4. [file 41598_2022_23198_MOESM4_ESM.gif]
